# Restriction-modification system with methyl-inhibited base excision and abasic-site cleavage activities

**DOI:** 10.1093/nar/gkv116

**Published:** 2015-02-19

**Authors:** Masaki Fukuyo, Toshiaki Nakano, Yingbiao Zhang, Yoshikazu Furuta, Ken Ishikawa, Miki Watanabe-Matsui, Hirokazu Yano, Takeshi Hamakawa, Hiroshi Ide, Ichizo Kobayashi

**Affiliations:** 1 Department of Medical Genome Sciences, Graduate School of Frontier Sciences, University of Tokyo, Tokyo 108-8639, Japan; 2 Institute of Medical Science, University of Tokyo, Minato-ku, Tokyo 108-8639, Japan; 3 Department of Evolutionary Studies of Biosystems, School of Advanced Sciences, The Graduate University for Advanced Studies (SOKENDAI), Hayama, Kanagawa 240-0193, Japan; 4 Department of Molecular Oncology, Graduate School of Medicine, Chiba University, Chiba 260-8670, Japan; 5 Department of Mathematical and Life Sciences, Graduate School of Science, Hiroshima University Higashi-Hiroshima 739-8526, Japan; 6 Department of Biophysics and Biochemistry, Graduate School of Science, University of Tokyo, Tokyo 113-8654, Japan

## Abstract

The restriction-modification systems use epigenetic modification to distinguish between self and nonself DNA. A modification enzyme transfers a methyl group to a base in a specific DNA sequence while its cognate restriction enzyme introduces breaks in DNA lacking this methyl group. So far, all the restriction enzymes hydrolyze phosphodiester bonds linking the monomer units of DNA. We recently reported that a restriction enzyme (R.PabI) of the PabI superfamily with half-pipe fold has DNA glycosylase activity that excises an adenine base in the recognition sequence (5′-GTAC). We now found a second activity in this enzyme: at the resulting apurinic/apyrimidinic (AP) (abasic) site (5′-GT#C, # = AP), its AP lyase activity generates an atypical strand break. Although the lyase activity is weak and lacks sequence specificity, its covalent DNA–R.PabI reaction intermediates can be trapped by NaBH_4_ reduction. The base excision is not coupled with the strand breakage and yet causes restriction because the restriction enzyme action can impair transformation ability of unmethylated DNA even in the absence of strand breaks *in vitro*. The base excision of R.PabI is inhibited by methylation of the target adenine base. These findings expand our understanding of genetic and epigenetic processes linking those in prokaryotes and eukaryotes.

## INTRODUCTION

**Figure 1.**
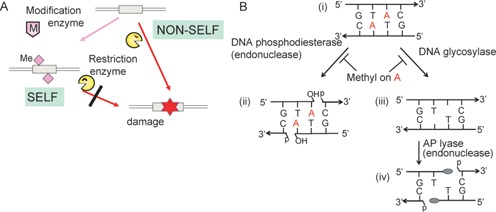
Restriction enzymes. (**A**) Restriction-modification systems. For a specific DNA sequence, an enzyme makes epigenetic modifications such as methylation to label self DNA. DNA without the modification is attacked by the paired restriction enzyme. Duplex lines, double-stranded DNA. Box, specific sequence. Magenta diamond, chemical modification such as methylation (Me) at the recognition sequence. (**B**) Two possible routes to DNA breakage for the restriction enzyme R.PabI. Top, a double-stranded DNA with a recognition sequence (i). Left, hydrolysis of phosphodiester bonds to generate two 3′-OH and 5′-P ends (ii). Right, generation of AP sites (iii). Cleavage generates two strand breaks (iv) with 5′-P and 3′-modified sugar (gray oval) ends. Red A, adenine to be excised unless methylated by a paired modification enzyme. See Supplementary Figure S4 for more detailed reaction mechanisms.

Restriction-modification (RM) systems recognize and attack nonself DNA based on DNA chemical modifications (Figure 1A). Among the various types of chemical modification, epigenetic methylation of bases is well studied. Methylation occurs at specific bases, generating m5C (5-methylcytosine, 5mC), m4C (N4-methylcytosine) or m6A (N6-methyladenine, mA) at specific sequences (1). RM systems are frequently encountered in the prokaryotic world and, less often, in the eukaryotic world (REBASE, http://rebase.neb.com) (2,3). These systems show mobility and variability in sequence recognition (4) and interact in cooperative or conflicting ways (5). RM systems have regulatory mechanisms reminiscent of mobile elements and toxin–antitoxin systems (6). Bacterial strains can have very different RM systems and methylomes, even genomic information indicates that they are closely related (7).

The biological significance of RM systems is not yet fully understood. RM systems were discovered through host controlled variation of bacterial viruses: a virus propagated in one host might not grow well in another host because of differences in DNA modification (8). RM systems attack incoming DNA such as viral genomes and transforming DNA as well as endogenous genomes with nonself epigenetic status (9,10). RM systems thus promote genetic isolation of a lineage. We hypothesize that they also drive adaptive evolution by introducing a specific global gene expression pattern (7,11–12).

All restriction enzymes examined so far hydrolyze phosphodiester bonds linking monomer nucleotide units leaving 3′-OH (hydroxyl) and 5′-P (phosphate) ends (Figure 1B (i, ii)). Thus, they are phosphodiesterases. A break on one strand is often coupled with a nearby break on the complementary strand, leading to a double-strand break. Restriction enzymes of Type II RM systems introduce a break at or near a specific DNA sequence unless the sequence is methylated at a specific base (1). Type II restriction enzymes have been important for molecular biology and genetic engineering (8). In addition, studies of their function and structure provide a paradigm for sequence-specific DNA–protein interaction (13,14).

Taking advantage of the mobility of Type II RM systems, we performed genome comparison and *in vitro* screening to identify a superfamily of restriction enzymes with a novel, half-pipe fold (15–17). The family includes R.PabI (restriction endonuclease of PabI RM system after the current nomenclature (1)) from *Pyrococcus*, a hyperthermophilic archaeon, and several from the Epsilonproteobacteria (16). R.PabI recognizes 5′-GTAC (16). The linked methyltransferase M.PabI (modification enzyme of PabI restriction-modification system) generates m6A in this sequence, which inhibits cleavage with R.PabI (18). A mesophilic eubacterial homolog of R.PabI (R.HpyAXII of *Helicobacter pylori*) limits transformation of DNA with an unmethylated form of this sequence (19).

In addition to the novel fold, R.PabI has several exceptional features: unlike most restriction enzymes examined, it does not require a divalent cation (17). It appeared to have generated a TA_OH_-3′ overhang (16). R.PabI DNA cleavage products examined by electrophoresis with single-base resolution showed bands that were broader than those generated with the neoschizomer R.RsaI (Figure 5 of (16)). This result suggested that DNA ends generated by R.PabI might be different from the 3′-OH and 5′-P ends. The product ends generated by purified R.PabI were difficult to religate, suggesting again that they did not have the typical 3′-OH and 5′-P end structures (Figure 1B (ii), see below). Our recent structural biology-based work demonstrated that R.PabI is a DNA glycosylase that excises an adenine base from the recognition sequence (Figure 1B (iii)) (20). This was unexpected because base excision has often been associated with repair of DNA damages. It also reminds of excision of a 5-methylcytosine base and its oxidized derivatives in DNA demethylation in plants and animals (21,22).

Finding of the base excision activity of the restriction enzyme immediately raises two questions. The first is about its relation to DNA strand breakage. The above work (20) concluded that the resulting AP (apurinic/apyrimidinic = abasic) site is transformed into a strand break by the β-elimination reaction at a high temperature in the (hyper)thermophilic bacteria or by a separate AP endonuclease in the mesophilic bacteria. A line of evidence for the latter route is the specific cleavage by the mixture of R.PabI and *Escherichia coli* lysate (20). This is consistent with specific cleavage with *E. coli* extract containing R.HpyAXII, a *H. pylori* homolog of R.PabI (19). The second question is its role in restriction. Which could be responsible for restriction phenomenon, base excision or strand breakage?

In the present work, we addressed these questions. We demonstrated a second activity in this enzyme that generates an atypical strand break at the AP site (Figure 1B (iv)). This cleavage is, however, not always coupled to the base excision. The base excision reaction turned out to be sufficient for restriction because the enzyme action impaired DNA biological activity in the absence of strand breakage *in vitro*.

## MATERIALS AND METHODS

### Bacterial strains, plasmids, viral genome and oligonucleotides

*Escherichia coli* strains and plasmids are in Supplementary Table S1. Genomic DNA of chlorella virus NY-2A (23) was kindly provided by Professor James Van Etten (University of Nebraska, Lincoln). Synthetic oligonucleotides used as enzyme substrates are in Supplementary Table S2.

### Expression of R.PabI

R.PabI with DNA cleavage activity was expressed in a strain co-expressing CviQI methyltransferase that generates 5′-GTm6AC-3′ as M.PabI (23). The M.CviQI coding region was PCR-amplified from genomic DNA of chlorella virus NY-2A with primers: M.CviQI forward primer : 5′-CGCGAATTCCCGTCGTGAGAATTTGCTCC-3′ and M.CviQI reverse primer: 5′-CGCAAGCTTGTCGTCCGCGGCATTGCTAT-3′. Amplified M.CviQI ORF was cut with EcoRI and HindIII and ligated with EcoRI and HindIII-cut pBAD30 (24) (Supplementary Table S1) by Ligation High (TOYOBO). Ligated samples were transformed into *E. coli* BL21(DE3) (25) by electroporation. The resulting strain-induced methylation of R.PabI recognition sites with arabinose (BYF25). Plasmid was designated pBAD30_*cviQIM* (Supplementary Figure S1A).

The R.PabI coding region was obtained from NdeI and BamHI-cut pMW40 constructed for expression *in vitro* (17). The fragment was ligated with NdeI and BamHI-cut pET28a (26) by Ligation High (TOYOBO). Ligated samples were transformed by electroporation as above into BYF25 competent cells from a culture with 0.5% arabinose, resulting in a strain that induced expression of R.PabI bearing HisTag with isopropyl β-d-1-thiogalactopyranoside (IPTG) in the presence of 0.5% arabinose (BYF72). The plasmid pET28a::*pabIR* was designated pYF46 (Supplementary Figure S1B).

### Purification of R.PabI

BYF72 cells were spread on LB agar plates with 50 μg/ml ampicillin, 50 μg/ml kanamycin and 0.5% arabinose. After overnight incubation at 37°C, single colonies were picked up and transferred to 5 ml LB with 50 μg/ml ampicillin, 50 μg/ml kanamycin and 0.5% arabinose. After overnight with shaking at 37°C, cultures were added to 1 l LB with 50 μg/ml ampicillin, 50 μg/ml kanamycin and 0.5% arabinose. After 7 h with shaking at 37°C, IPTG was added to a 1 mM final concentration with incubation at 37°C for 4 h followed by centrifugation at 3.5 krpm for 10 min at 4°C. Cells were resuspended in 60 ml of 20 mM Tris–HCl (pH7.5) with 150 mM NaCl and divided into 30 ml aliquots that were sonicated (Ultrasonic Disruptor UD-200, TOMY) and centrifuged at 7 krpm for 20 min at 4°C. Supernatants were heated at 75°C for 10 min followed by centrifugation at 7 krpm for 20 min at 4°C.

R.PabI was purified from supernatants through three columns. Supernatants were treated with HisTrap Kits (Amersham Biosciences) with a buffer change by dialysis with 10 mM Tris–HCl (pH7.5), 100 mM NaCl and 1 mM dithiothreitol (DTT). Enzymes were purified with heparin Sepharose CL-6B (Amersham Biosciences) followed by dialysis with 20 mM Tris–HCl (pH7.5), 150 mM NaCl and 2.5 mM CaCl_2_. Histidine-tagged R.PabI was digested with thrombin (Novagen) at 20°C for 16 h. Tag-free R.PabI were obtained by the following purification with benzamidine Sepharose 6B (Amersham Biosciences) and dialysis against 10 mM 2-(N-morpholino)ethanesulfonic acid (MES) (pH 6.0), 100 mM NaCl, 0.1 mM ethylenediaminetetraacetic acid (EDTA), 1 mM DTT and 50% glycerol. Resulting R.PabI preparations showed a single band by SDS-PAGE with the expected molecular weight as calculated from the amino acid sequence. From 1 l of *E. coli* culture, 400 μg of purified R.PabI was obtained. Storage of R.PabI in the above solution (10 mM MES (pH 6.0), 100 mM NaCl, 0.1 mM EDTA, 1 mM DTT and 50% glycerol) at −80°C for 4 years has not resulted in noticeable changes to DNA cleavage activity.

**Figure 2.**
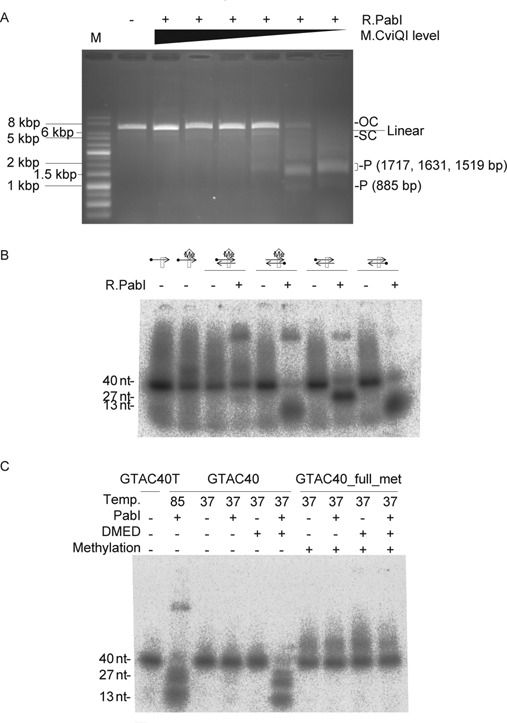
Inhibition of R.PabI activities by methylation. (**A**) Inhibition of strand cleavage. Plasmid pBAD30_*cviQIM* (Supplementary Figure S1) with a gene for M.CviQI, which generates 5′-GTm6AC as M.PabI does, under control of the pBAD promoter was prepared from cultures with varying concentrations of arabinose, its inducer. After incubation with R.PabI at 85°C for 6 h, the plasmid DNAs were subjected to 0.8% agarose gel electrophoresis. OC, open circle; SC, supercoiled; P, product DNA. Left lane: 1 kb DNA Ladder. (**B**) Strand-specific inhibition of cleavage in hemimethylated double-stranded DNA. A 40-mer single-stranded (GTAC40T or GTAC40Tme, Supplementary Table S2) or double-stranded (GTAC40_hemi_met or GTAC40, Supplementary Table S2) substrate (1 pmol, 100 nM) with a ^32^P-label (black dot) at the 5′-end of either strand was incubated with R.PabI (9.2 pmol, 920 nM) at 85°C for 3 h. Products were separated by 10% denaturing PAGE. Box, recognition sequence (5′-GTAC). Me diamond, methylation of the top strand. Cleavage at the recognition sequence resulted in 27-mer and 13-mer oligonucleotides. The supershifted bands near the top of the gel are likely DNA–R.PabI complexes (see also Figure 5 and related text). (**C**) Inhibition of DNA glycosylase. A 40-mer double-stranded substrate (GTAC40 or GTAC40_full_met (Supplementary Table S2), 1 pmol, 100 nM) with a ^32^P -label at the 5′ end of both strands was incubated with R.PabI (9.2 pmol, 920 nM) and then treated with 0.1 M DMED at 37°C for 1 h. Products were separated by 10% denaturing PAGE.

**Figure 3.**
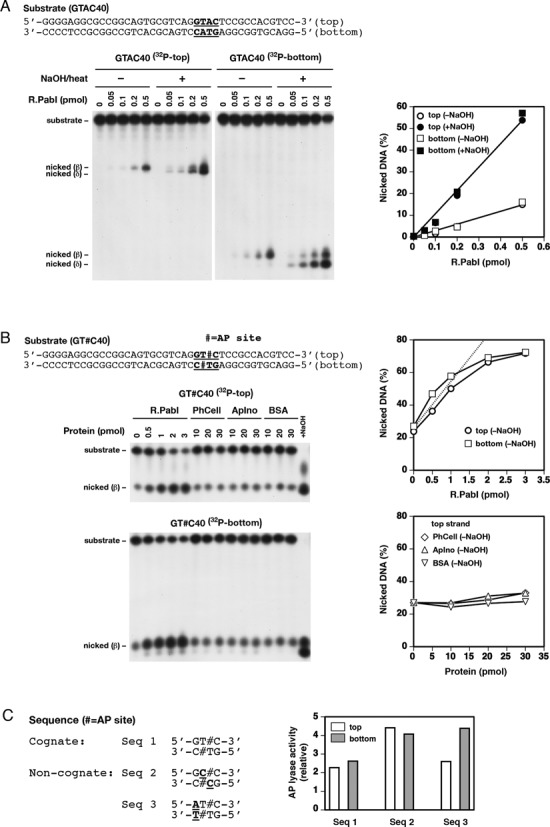
R.PabI has DNA glycosylase and uncoupled AP lyase activities. (**A**) DNA glycosylase activity. A 40-mer double-stranded substrate (GTAC40 (Supplementary Table S2), 0.2 pmol, 10 nM) with a 5′-^32^P label on the top or bottom strand was incubated with R.PabI (0–0.5 pmol, 0–25 nM) at 70°C for 1 h. After incubation, half the reaction mixture was treated with 0.1 M NaOH at 70°C for 10 min to cleave AP sites. Left, samples separated by 18% denaturing PAGE. Right, amount of nicked DNA without (–) or with (+) postreaction NaOH treatment. Plots with +NaOH show DNA glycosylase activity. (**B**) AP lyase activity. Reactions were performed as in (A) except that the substrate (GT#C40, Supplementary Table S2) contained two AP sites at the indicated positions and no postreaction NaOH treatment was included. The substrate (0.2 pmol, 10 nM) was incubated with R.PabI (0–3 pmol, 0–150 nM), *Pyrococcus horikoshii* OT3 cellulase (PhCell), *Aeropyrum pernix* K1 inositol 1-phosphate synthase (ApIno), or BSA (all 0–30 pmol, 0–1500 nM) at 70°C for 1 h. Products were analyzed by 18% denaturing PAGE (gels on the left). The amounts of nicked DNA for top and bottom strands are plotted in graphs on the right. Note that ∼25% of the DNA substrate underwent spontaneous cleavage at AP site without enzyme treatment during reactions. The dotted line in the upper graph for R.PabI is a linear regression of the initial averaged slopes of top and bottom strand cleavage. The lower graph for proteins other than R.PabI shows the data for the top strand cleavage alone for clarity. The result for the bottom strand cleavage was similar to that for the top strand. (**C**) Sequence specificity of AP lyase activity. Reactions of R.PabI (0–1 pmol, 0–50 nM) were performed as in (B) using 40-mer double-stranded substrates (GT#C40, GC#C40, and AT#C40 (Supplementary Table S2), 0.2 pmol, 10 nM) containing two AP sites in the indicated sequence contexts (Seq 1–3). The activities for AP sites in Seq 1–3 were determined from the relationship between the amounts of R.PabI and nicked products (after subtraction of spontaneous cleavage) and the relative activities are plotted against Seq 1–3 in the graph. All graph data in (A)–(C) are the average of two independent experiments.

**Figure 4.**
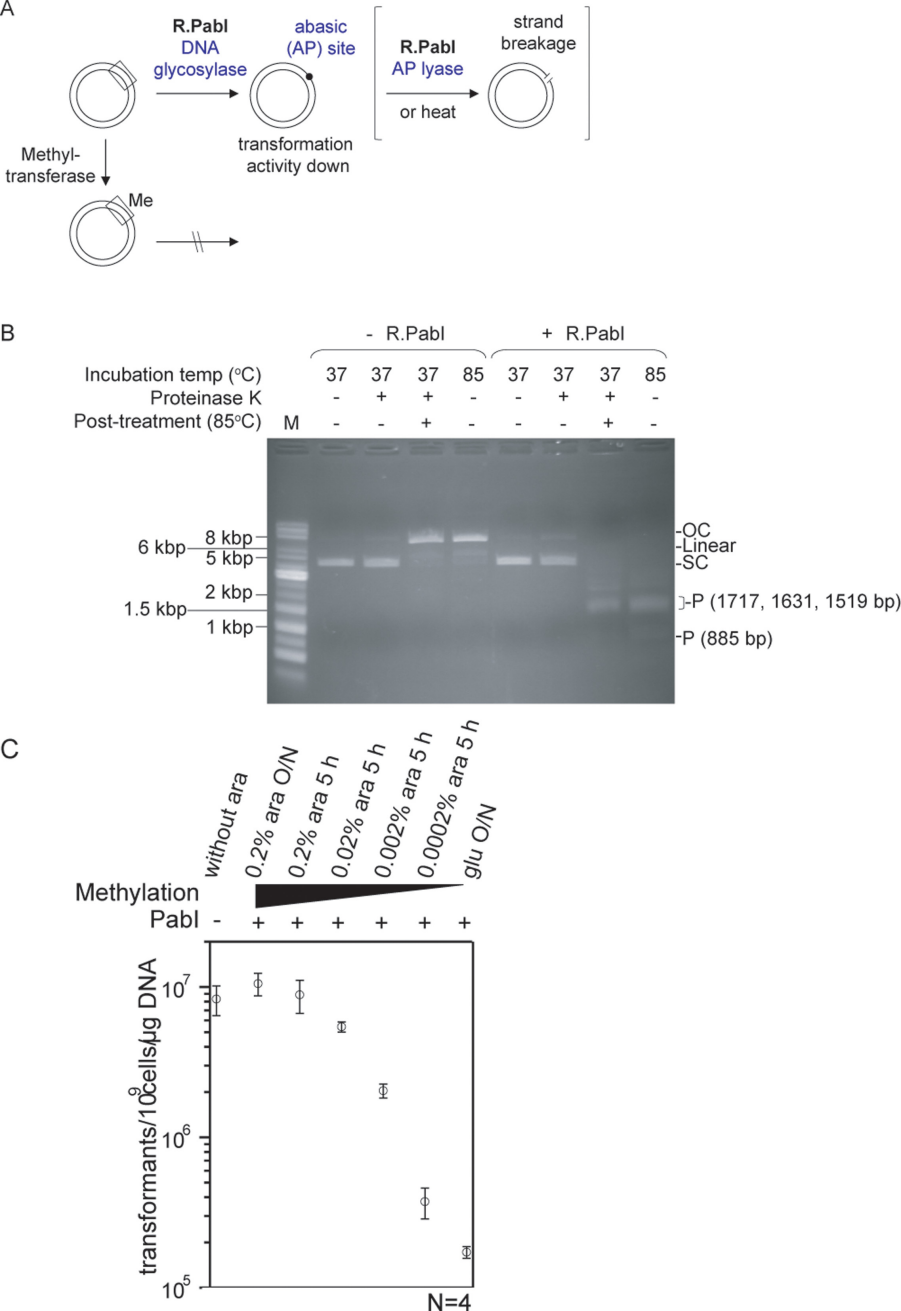
R.PabI restricted DNA without strand breaks. (**A**) Design. Double-stranded, circular plasmid DNA was treated with R.PabI and transformation activity is measured. Box, recognition sequence. Me, base methylation within the recognition sequence. Only one recognition sequence is shown for simplicity. (**B**) R.PabI treatment at 37°C does not cause single-strand breaks. Plasmid pBAD30_*cviQIM* (0.11 pmol, 11 nM, Supplementary Figure S1) purified from *E. coli* under noninducing conditions was incubated with or without R.PabI (0.77 pmol, 77 nM) at 37°C for 30 min, which was followed by proteinase K (ProK) treatment at 37°C overnight to inactivate R.PabI. DNA was incubated at 85°C for 6 h. Alternatively, plasmid was incubated with or without R.PabI at 85°C for 6 h. Samples were subjected to 0.8% agarose gel electrophoresis. OC, open circle; SC, supercoiled circle; P, product DNA. Left lane: 1 kb DNA ladder. (**C**) Loss of DNA transformation after R.PabI treatment. pBAD30_*cviQIM* (0.11 pmol, 11 nM, Supplementary Figure S1) with varying levels of methylation was treated with R.PabI (0.77 pmol, 77 nM) at 37°C for 30 min and purified for quantitative transformation (‘Materials and Methods’ section, Supplementary Figure S5) of *E. coli*. The plasmid was prepared from cultures with 0.2% arabinose overnight, 0.2% arabinose 5 h, 0.02% arabinose 5 h, 0.002% arabinose 5 h, 0.0002% arabinose 5 h, and glucose overnight, in the order of expected decreased methylation level. *E. coli* HST08 was transformed with R.PabI-treated plasmids, and resulting colonies were counted to determine transformation efficiencies.

### Preparation of methylated plasmid DNAs (Figures 2A and 4C)

A single colony of *E. coli* BMF235 ( = HST08 (pBAD30 *cviQIM*), Supplementary Table S1) was cultivated in LB broth with 0.0002, 0.002, 0.02 or 0.2% arabinose and 100 μg/ml ampicillin with aeration at 37°C. Culture containing 20 mM glucose instead of arabinose was prepared as a negative control to repress expression of the *cviQIM* gene. After 5 h or one overnight, plasmid DNA was purified from 5 ml culture using GenElute Plasmid Miniprep Kits (Sigma–Aldrich).

### ^32^P-labeling of oligonucleotides and preparation of duplexes

**Figure 5.**
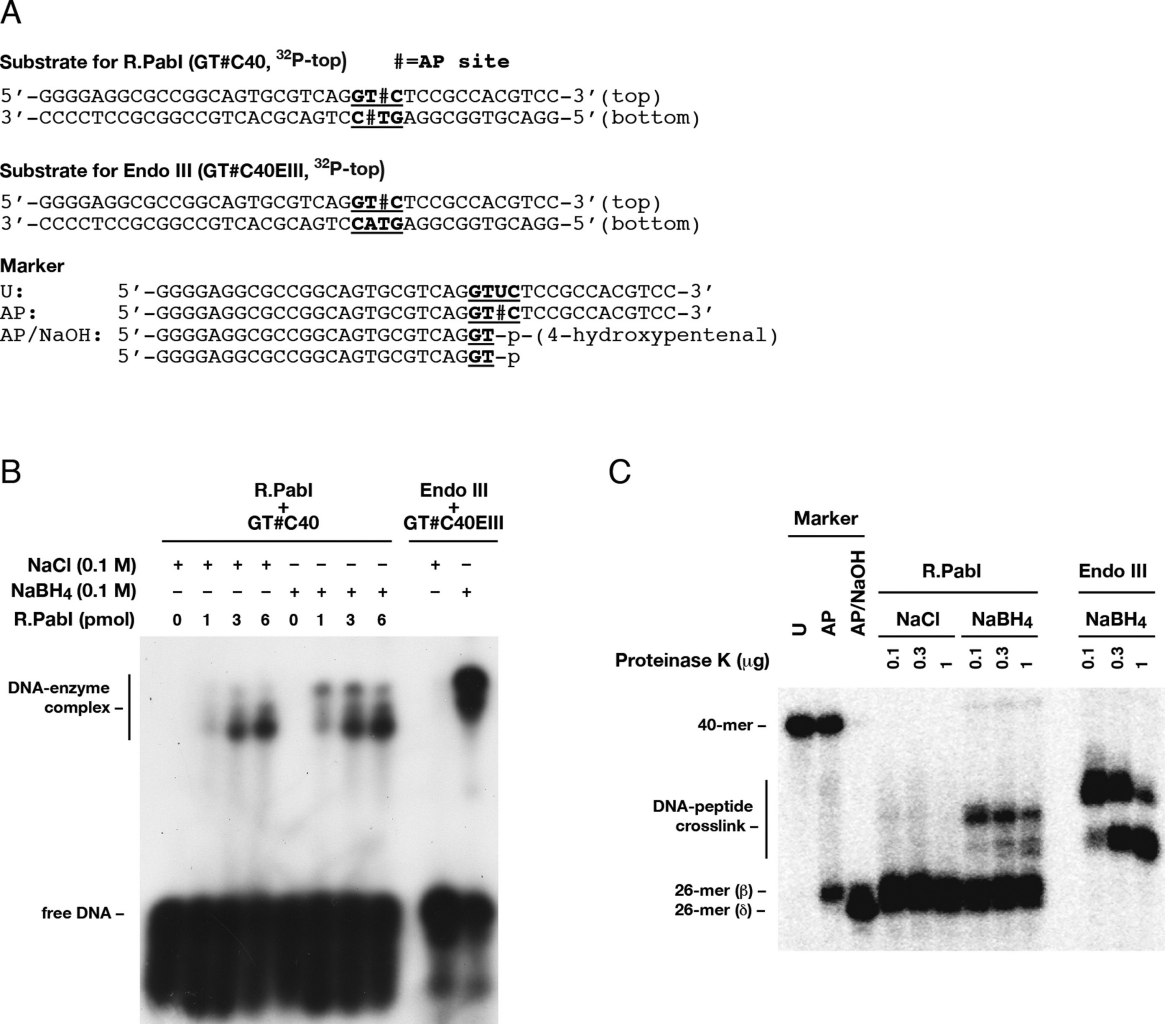
R.PabI forms Schiff-base intermediates. (**A**) Sequences of substrates and markers used for analysis of reaction intermediates. (**B**) Analysis of DNA–R.PabI complexes. R.PabI (0–6 pmol, 0–300 nM) and a double-stranded substrate (GT#C40 (Supplementary Table S2) with a 5′-^32^P label in the top strand, 0.2 pmol, 10 nM) were incubated at 70°C for 20 min and then with 0.1 M NaBH_4_ (or 0.1 M NaCl) at 25°C for 30 min. The reaction mixture was mixed with a gel loading buffer (final SDS concentration = 3%), denatured at 90°C for 10 min, and separated by 10% SDS-PAGE. Endo III (20 units) was incubated with a substrate (GT#C40EIII (Supplementary Table S2) with a 5′-^32^P label in the top strand, 0.2 pmol, 10 nM) at 37°C for 20 min and subjected to NABH_4_-trapping. (**C**) Analysis of covalently-trapped intermediates. NaBH_4_-trapping reactions of R.PabI and Endo III were performed as in A (5-fold scale relative to A). DNA–enzyme complexes were separated by SDS-APGE, and gel bands containing DNA–enzyme complexes (corresponding to bands marked as DNA–enzyme complexes in B) were excised. DNA–enzyme complexes were electro-eluted from the gel (multiple bands altogether), desalted by a spin column, and treated with the indicated amounts of proteinase K. The resulting products were analyzed by 18% denaturing PAGE. The bands indicated as a DNA–peptide crosslink for R.PabI and Endo III likely correspond to (xi) in Supplementary Figure S4C except that R.PabI and Endo III proteins were digested into short peptides of different sizes.

Reactions were 100 pmol single-stranded oligonucleotides, 10 units of T4 polynucleotide kinase (TaKaRa), 740 kBq [γ-^32^P]ATP (PerkinElmer), 50 mM Tris–HCl, 10 mM MgCl_2_, 5 mM DTT in 10 μl at 37°C for 30 min followed by kinase inactivation at 90°C for 2 min and purification with MicroSpin G-25 columns (GE Healthcare) (experiments in Figure 2BC). Alternatively, single-stranded oligonucleotides were labeled similarly and purified by Centri-Sep Spin columns (Princeton Separations) (experiments in Figures 3 and 5). For double-stranded oligonucleotide substrates, equimolar amounts (5 pmol each) of a single-stranded oligonucleotide and its complement (Supplementary Table S2) were incubated at 96°C for 5 min and cooled to room temperature.

### DNA cleavage assays

Reactions for plasmids were 10 μl with 0.77 pmol (77 nM) of R.PabI and 0.11 pmol (11 nM) of plasmid DNA in 0.1 M sodium phosphate buffer (pH 6.5) at 85°C for 6 h. DNA was separated by agarose gel electrophoresis and visualized with GelRed Nucleic Acid Gel Stain (Biotium) and ultraviolet light (experiments in Figure 2A). For cleavage reactions of oligonucleotides, 1 pmol (100 nM) of a oligonucleotide substrate was reacted with 9.2 pmol (920 nM) of R.PabI in 10 μl of 0.1 M sodium phosphate buffer (pH 6.5) at 85°C for 3 h. Products were separated at 50 V for 2 h through 10% Long Ranger polyacrylamide gels (TaKaRa) (experiments in Figure 2B).

### DNA glycosylase assays

Reactions for plasmids were 10 μl with 0.77 pmol (77 nM) of R.PabI, 0.11 pmol (11 nM) of plasmid DNA in 0.1 M sodium phosphate buffer (pH 6.5), incubated at 37°C for 30 min. As indicated, reaction mixtures were treated with 5 μg/ml proteinase K in10 mM EDTA and 0.1% sodium dodecyl sulfate (SDS) overnight at 37°C and incubated at 85°C for 6 h (experiments in Figure 4B). In experiments with oligonucleotides, 1 pmol (100 nM) of a double-stranded oligonucleotide substrate was reacted with 9.2 pmol (920 nM) of R.PabI in 10 μl of 0.1 M sodium phosphate buffer (pH 6.5) at 37°C for 3 h and purified with NucleoSpin Extract II Kits (MACHEREY-NAGEL). Half the reaction mixtures were treated with 0.1 M *N*,*N*′- dimethylethylenediamine (DMED) at 37°C for 1 h to cleave DNA at AP sites. Products were separated through 10% Long Ranger polyacrylamide gels (TaKaRa) by 50 V for 2 h (experiments in Figure 2C).

Alternatively, 0.2 pmol (10 nM) of a double-stranded oligonucleotide substrate containing the 5′-GTAC sequence and a 5′-^32^P label on either strand was incubated with 0–0.5 pmol (0–25 nM) of R.PabI in 0.1 M phosphate buffer (pH 6.5, 20 μl) at 70°C for 1 h. Half the reaction mixture was treated with 0.1 M NaOH at 70°C for 10 min to cleave DNA at AP sites and neutralized with HCl. Samples were separated by 18% denaturing PAGE (experiments in Figure 3).

### AP lyase assay (Figure 3BC)

Adenine nucleotides in the recognition sequence (5′-GTAC/3′-CATG) were replaced by AP sites by incubating uracil-containing (5′-GTUC/3′-CUTG) double-stranded oligonucleotides (Supplementary Table S2) with uracil N-glycosylase (UNG) (New England Biolabs). Complete conversion to AP sites was confirmed by NaOH cleavage.

AP lyase reactions were performed in the presence of 0–3 pmol (0–150 nM) of R.PabI as described above for DNA glycosylase reactions at 70°C for 1 h except that the substrate duplexes (0.2 pmol, 10 nM) contained two AP sites and no postreaction NaOH treatment was included. To confirm that R.PabI has an intrinsic (i.e., enzymatic) AP lyase activity, 0.2 pmol (10 nM) of the substrate duplex containing two AP sites was incubated with 0–30 pmol (0–1500 nM) of bovine serum albumin (BSA) or a thermophylic enzyme (*Pyrococcus horikoshii* OT3 cellulase and *Aeropyrum pernix* K1 inositol 1-phosphate synthase; both were generous gifts from Thermostable Enzyme Laboratory, Co.), and the resulting products were analyzed as described for the AP lyase activity of R.PabI. The sequence specificity of R.PabI AP lyase activity was analyzed using 0–1 pmol (0–50 nM) of R.PabI and 0.2 pmol (10 nM) of the substrate duplexes containing two AP sites in cognate and non-cognate sequences (Supplementary Table S2).

### NaBH_4_ trapping (Figure 5)

R.PabI (0–6 pmol, 0–300 nM) and a double-stranded substrate DNA (0.2 pmol, 10 nM) containing 5′- GT#C (# = AP site) and a 5′-^32^P label on the top strand were incubated in 0.1 M phosphate buffer (pH 6.5, 20 μl) at 70°C for 20 min, and then with 100 mM NaBH_4_ (or 100 mM NaCl) at 25°C for 30 min. DNA–R.PabI complexes were denatured in gel loading buffer containing 3% SDS at 90°C for 10 min and separated by 10% SDS-PAGE. For controls, 20 units of Endo III (New England Biolabs) and 0.2 pmol (10 nM) a double-stranded substrate containing an AP site and a 5′-^32^P label on the top strand was incubated in 10 mM Tris–HCl (pH 7.5), 100 mM NaCl and 1 mM EDTA (total 20 μl) at 37°C for 20 min followed by 100 mM NaBH_4_ (or 100 mM NaCl) as above. DNA–Endo III complexes were analyzed as described for R.PabI.

In addition, NaBH_4_-trapping reactions were also performed in a large scale (1 pmol of substrate DNA and 30 pmol of R.PabI or 100 units of Endo III). DNA–enzyme complexes were separated by 10% SDS-PAGE, electro-eluted from the gel bands, concentrated by evaporation, and desalted by Centri-Sep Spin columns. DNA–enzyme complexes (ca. 0.05 pmol) were digested with 0.1–1 μg of proteinase K (Wako) and separated by 18% denaturing PAGE.

### Quantitative transformation (Figure 4C)

Reactions of 10 μl contained 0.77 pmol (77 nM) of R.PabI and 0.11 pmol (11 nM) of plasmid DNA in 0.1 M sodium phosphate buffer (pH 6.5). After 0.5 h at 37°C, plasmid DNA was purified with a NucleoSpin Extract II (MACHEREY-NAGEL) and 0.11 pmol (200 ng) of plasmid DNA was mixed with a competent cell suspension with ∼10^8^ bacteria (*E. coli* HST08 Premium Competent Cells, TaKaRa; Supplementary Table S1) thawed on ice. The mixture was kept on ice for 15 min, at 42°C for 60 s, and on ice for 5 min, and then 600 μl of SOC medium (2% tryptone, 0.5% yeast extract, 10 mM NaCl, 2.5 mM KCl, 10 mM MgSO_4_, 10 mM MgCl_2_, 20 mM glucose; TaKaRa) was added to the mixture. Cells were incubated for 60 min at 37°C. Serial dilutions of the cell culture were spread on LB plates (1% tryptone, 0.5% yeast extract, 1% NaCl, 1.3% agar) containing 100 μg/ml ampicillin.

## RESULTS

### Expression of R.PabI *in vivo* with chlorella virus DNA methyltransferase

Expression of R.PabI in *E. coli* was difficult, probably because of its toxic effect on the genome. We earlier employed an *in vitro* expression system to determine the R.PabI crystal structure (17). We also coexpressed the cognate modification enzyme M.PabI, which generates 5′-GTm6AC (18). M.PabI overexpression protects chromosomal DNA from digestion by R.RsaI, which recognizes the same target sequence as R.PabI. However, we could not establish a plasmid carrying an R.PabI gene with a bacterial translation signal in the strain overexpressing M.PabI. The purified M.PabI shows hyperthermophilic activity with a maximum at 85°C and a minimum at 35°C (18). Therefore, we used the modification enzyme M.CviQI from chlorella virus NY-2A (23) expressed from an arabinose-inducible promoter to introduce 5′-GTm6AC into the host genome (Supplementary Figure S1A). In the presence of M.CviQI, a plasmid carrying the R.PabI gene was established, allowing overproduction of the enzyme. We purified R.PabI in a tag-free form (‘Materials and Methods’ section) to a single band in SDS-PAGE consistent with its expected molecular weight (26 kDa).

### Features of R.PabI-mediated DNA cleavage

Cleavage by R.PabI at 85°C of a plasmid carrying the gene for chlorella methyltransferase (M.CviQI) was inhibited by expression of the methyltransferase before plasmid preparation (Figure 2A) as expected (18). Methylation sensitivity was confirmed with a 40 bp synthetic double-stranded oligonucleotide with a methylated adenine in the recognition sequence (5′-GTmAC) (Supplementary Figure S2). When the double-stranded DNA was hemimethylated, only the unmethylated strand was cleaved (Figure 2B). In the presence of R.PabI, upper shifted bands were also visible (Figure 2B). These bands are likely DNA–R.PabI complexes (see also Figure 5 and related text for further information). The very tight binding of R.PabI to DNA even under denaturing conditions further suggests a low turnover rate of R.PabI.

The ligation efficiency for DNA fragments produced by R.PabI was very low (Supplementary Figure S3), while blunt ends generated by R.RsaI (…GT_OH_-3′ and 5′-_P_AC…) were efficiently ligated. This result suggested that R.PabI cleavage produced DNA-end structures that were not suitable substrates for the DNA ligase. We came to doubt whether they were 3′-OH and 5′-P ends, shared by all the restriction endonucleases examined (see also ‘Introduction’ section).

### R.PabI has DNA glycosylase activity inhibited by methylation

Our recent structural biology-based work demonstrated that R.PabI acts as a DNA glycosylase (also known as DNA N-glycosylase or DNA N-glycosidase) that excises an adenine base in its recognition sequence (5′-GTAC) even at a low temperature (40°C) (20) (Supplementary Figure S4). Because the resulting AP sites will generate strand breaks after heat treatment (27), this finding is consistent with the features of R.PabI-mediated cleavage at the high temperature. These include independence of divalent metal cation (17) and the electrophoretic mobility of the cleavage products like those with 5′-GTA_OH_ and _P_C-3′ ends (16).

The excised adenine is the base to be methylated by the cognate methyltransferase M.PabI (18) and chlorella M.CviQI (23). The crystal structure can provide an explanation as to how such methylation can prevent the excision and hence the breakage (20). We here examined whether the methylation does inhibit the glycosylase. To detect base excision, we used a standard DNA glycosylase assay based on alkali lability of AP sites. A synthetic double-stranded DNA containing the 5′-GTAC recognition sequence was incubated with R.PabI at 70^o^C and products were analyzed by denaturing PAGE (Figure 3A). A single-strand break was observed in both strands without postreaction NaOH treatment and increased with postreaction NaOH treatment. Comparable amounts of AP sites were produced for the top and bottom strands, indicating that R.PabI glycosylase activity acted on both strands, similar to the phosphodiesterase activity of dimeric restriction enzymes that recognize a palindromic sequence. This result was consistent with dimerization of R.PabI in crystal and solution (17) and the double-strand breaks at the recognition sequence of R.PabI-treated plasmid DNA upon heating (Figure 4B). Generation of AP sites was confirmed further by DMED treatment, which specifically cleaves DNA strand at AP sites (28) (Figure 2C). Glycosylase activity was not observed with methylated synthetic DNA (Figure 2C, Supplementary Figure S2). The upper shifted band observed in the second lane is likely the DNA–R.PabI complex.

Taken together, these results suggested that the methylation-mediated inhibition of strand breakage resulted from preventing the base excision.

### R.PabI has AP lyase activity

DNA glycosylases are classified as bifunctional if they have an associated AP lyase activity that introduces a break at the AP site (Supplementary Figure S4C) (29,30). To determine if R.PabI was bifunctional, the enzyme was incubated with double-stranded DNA with two AP sites lacking the adenine in the recognition sequence (Figure 3B). Products were analyzed by denaturing PAGE without postreaction NaOH treatment. The substrate DNA was ∼25% cleaved without R.PabI because of the intrinsic instability of AP sites under the 70°C, 1 h condition. However, addition of R.PabI promoted incision of the AP sites at both strands (Figure 3B), demonstrating that R.PabI had AP lyase activity. In contrast, addition of BSA or thermophlic enzymes (cellulase and inositol 1-phosphate synthase) up to 10-fold more than R.PabI did not result in an increase in the amount of products (Figure 3B), indicating that the incision activity of R.PabI for AP sites was not due to the non-enzymatic reaction of proteins but was the enzymatic activity of R.PabI.

The glycosylase and AP lyase activities of R.PabI were not coupled because most AP sites generated by the glycosylase remained uncleaved in the absence of NaOH treatment (Figure 3A). R.PabI had greater activity on the intact cognate substrate (5′-GTAC) as glycosylase than the substrate with the AP site (5′-GT#C, # = AP site) as AP lyase (Figure 3AB). One pmol of R.PabI produced 0.21 pmol of AP sites by glycosylase activity (intact substrate) and 0.057 pmol of incised AP sites by AP lyase activity (AP site-containing substrate, data calculated from the initial slope for the fitting line in Figure 3B), with an apparent ratio of glycosylase versus AP lyase activities of 3.7:1. The DNA glycosylase and AP lyase activities of R.PabI increased with increasing reaction temperature (data not shown). This observation explains the temperature-dependence of the R.PabI's double-strand DNA cleavage activity (16) and is in harmony with the fact that R.PabI is an enzyme from the hyperthermophilic archaeon, *Pyrococcus*.

The sequence specificity of R.PabI AP lyase was analyzed using substrates containing two AP sites in sequence contexts other than GTAC (Figure 3C). R.PabI incised AP sites embedded in cognate (GT#C, # = AP site) and non-cognate (GC#C, AT#C, and GT#T, # = AP site) sequences with a comparable efficiency. Thus, the sequence specificity of the AP lyase of R.PabI was markedly relaxed compared to the DNA glycosylase activity of R.PabI, which was shown to be very stringent in a previous study (20).

### R.PabI forms Schiff base intermediates with DNA

A possible scenario of the uncoupling of the glycosylase and AP lyase (preceding section) is initial release of adenine by substitution with water (glycosylase reaction) (Supplementary Figure S4A) and subsequent opportunistic Schiff-base formation between the resulting AP site and R.PabI (Supplementary Figure S4C (vii, ix)), leading to partial cleavage of the AP sites (Supplementary Figure S4C (x, xiii)). Schiff-base intermediates characteristic of bifunctional DNA glycosylases (Supplementary Figure S4C (vii, ix)) can be stabilized by reduction with NaBH_4_ (Supplementary Figure S4C (viii, xi)) (29–31). Therefore, the reaction mixture of R.PabI and the DNA substrate containing two AP sites were treated with NaBH_4_ (or NaCl for control) and the products were analyzed by SDS-PAGE after heat denaturation in gel loading buffer containing 3% SDS (Figure 5AB). Both of treatment with NaBH_4_ and NaCl resulted in shifted bands indicative of DNA–R.PabI complexes (Figure 5B), and the amount of the complexes was slightly more for NaBH_4_ than NaCl. This result indicated that non-covalent DNA–R.PabI complexes were resistant to denaturation with heat and SDS and that non-covalent and covalent complexes could not be distinguished by SDS-PAGE. Conversely, non-covalent and covalent DNA–Endo III complexes were clearly distinguished by SDS-PAGE (Figure 5B).

To distinguish between the non-covalent and covalent DNA–R.PabI complexes, trapping reactions were performed in a large scale, and the resulting DNA–R.PabI complexes were separated by SDS-PAGE and eluted from gel bands in a lump (i.e. multiple bands altogether). The purified complexes were digested with proteinase K and the resulting products were analyzed by denaturing PAGE (Figure 5C). The NaCl treatment resulted in only free DNA (26-mer) arising from the spontaneous cleavage at the AP site, whereas the NaBH_4_ treatment resulted in shifted bands indicative of DNA (26-mer)-peptide crosslinks, together with free 26-mer products. This result indicated that NaBH_4_-treated DNA–R.PabI complexes indeed contained covalent DNA–R.PabI complexes (Schiff base) together with non-covalent complexes. The DNA-tethered R.PabI-derived peptides generated after digestion with 0.1 μg of proteinase K (upper major band in Figure 5C, left, lane 7) were converted partly to shorter peptides (lower bands in Figure 5C, left, lanes 8 and 9) upon more extensive digestion with proteinase K (0.3 and 1 μg). The digestion of DNA–Endo III complexes, which were also purified from the SDS-PAGE gel bands, afforded crosslinked products containing peptides of two different sizes (Figure 5C). The absence of free 26-mer DNA for Endo III indicated that non-covalent DNA–Endo III complexes, if present, were dissociated completely during SDS-PAGE separation.

### Restriction activity in the absence of DNA strand breaks

The aforementioned results clearly demonstrated that R.PabI has two enzymatic activities: DNA glycosylase and AP lyase. R.PabI restriction mechanism could be different from DNA phosphodiester bond hydrolysis. Which of these activities, base excision and strand breakage, are important in the restriction process? We examined whether R.PabI impairs DNA activity in the absence of DNA strand breaks (Figure 4A). We used circular double-stranded plasmid DNA molecules. When they have no strand breaks, they are supercoiled and migrate at a specific rate in agarose gel electrophoresis. A single-strand break would result in an open circle with a different migration rate. As a measure of DNA activity, we used the quantitative transformation assays for plasmids (Supplementary Figure S5). Note here that our definition of restriction is based on inhibition of propagation of a genome, as explained in ‘Introduction’ section, rather than on DNA cleavage.

Plasmid DNA treated with R.PabI at a low temperature (37°C) mainly remained supercoiled (Figure 4B, fourth lane from the right). This indicates that most of the treated DNAs lack any strand breaks. However, this treatment decreased transformation efficiency by two orders of magnitude (Figure 4C, the leftmost point and the rightmost point). This inactivation was inhibited by the cognate methylation (Figure 4C, right to left). These results indicated that the PabI RM system acted through a mechanism other than strand breakage *in vitro*.

When DNA treated with R.PabI at low temperature was purified to remove R.PabI and heat treated, double-strand breaks were detected at the recognition sites (Figure 4B). Heat-induced double-strand breaks were not detected in DNA not pretreated with R.PabI, and only single-strand breaks were observed (Figure 4B). These results suggested that the DNA damage generated by R.PabI was a precursor form of double-strand breaks at the recognition sequence. It is likely to be base excision. The DNA damage that (i) led to decreased biological activity, (ii) did not involve strand breakage, (iii) was inhibited by adenine methylation in the recognition sequence (5′-GTAC), and (iv) led to double-strand breaks at the recognition sequence after heat treatment, as demonstrated above, is likely to be excision of the adenine base from both strands at the recognition sequence. In other words, DNA glycosylase activity can promote restriction in this RM system.

## DISCUSSION

### Novel class in restriction enzymes and restriction modification systems

Our results demonstrated that R.PabI restricted the biological activity of DNA in the absence of strand breaks through glycosylase activity. Because this restriction was inhibited by methylation of a specific base in the enzyme's recognition sequence, this represents a novel mode in restriction modification processes. We also demonstrated that R.PabI has AP lyase activity, which acted on the resulting AP site and generated a strand break, although the lyase activity is weak and lacks sequence specificity. These two activities are consistent with the DNA double-strand cleavage activity seen in crude extracts from *E. coli* containing *H. pylori* homolog of R.PabI at a low temperature (37°C) (19), although possible contribution of breakage at the AP sites occurring spontaneously or mediated by *E. coli* enzymes cannot be excluded. We do not know whether *in vivo* restriction of DNA with the AP sites (Figure 4C) requires strand breakage by an AP lyase (of R.PabI or of the recipient *E. coli*) or an AP endonuclease (of the recipient *E. coli*). Although the presence of bi-stranded clustered AP sites on DNA *in vivo* is known to affect cellular processes involving DNA replication and repair (32,33), we do not know anything about the processing *in vivo* of AP sites generated by R.PabI *in vitro*.

Based on the two activities of R.PabI described here, we propose classifying restriction enzymes into the *restriction phosphodiesterase* class and the *restriction glycosylase/lyase* class. The former class would include all restriction enzymes that act through the hydrolysis of phosphodiester bonds; the latter class would be the PabI superfamily with the half-pipe fold (17), including R.PabI and its homologs in Epsilonproteobacteria such as *Helicobacter* and *Campylobacter* (16–17,20). We would like to use the same classification for restriction modification systems. This classification should replace their classification into the restriction endonucleases and the restriction glycosylases in our previous publication (20) because the AP lyase acts as an endonuclease.

### Reaction mechanisms

Bifunctional DNA glycosylases release a base and break a strand in a concerted manner that leaves no uncleaved AP sites. An initial attack on the sugar C1’ by a nucleophilic residue causes scission of the N-glycosidic bond and concomitant formation of a covalent Schiff base intermediate DNA–enzyme complex (Supplementary Figure S4C, (vii)) (29,30). Subsequent β-elimination of the 3′-P (phosphate) group leads to strand scission (Supplementary Figure S4C, (ix)).

The AP lyase activity of R.PabI was not coupled to its glycosylase activity (Figure 3A). Unlike bifunctional DNA glycosylases, R.PabI catalyzed initial base release and β-elimination in separate reactions. The initial base release likely involved substitution of the adenine by a water molecule, which is characteristic of the monofunctional DNA glycosylases (Supplementary Figure S4AB). Subsequent rearrangement of the DNA or the active site residues of R.PabI might result in the formation of the Schiff-base intermediate that leads to strand scission via β-elimination (Supplementary Figure S4C).

Uncoupling of glycosylase and AP lyase activities has been reported for the bacterial MutY glycosylase that removes adenine from an adenine:8-oxoguanine pair (34), the human OGG1 glycosylase that removes 8-oxoguanine from an 8-oxoguanine:cytosine pair (35,36), human Nth (hNth) that removes pyrimidines modified by reactive oxygen species (37), and mouse Nei homolog MmuNeil3 that removes oxidation products of 8-oxo-G paired with C (38). For MutY, two distinct but closely related mechanisms have been proposed for the glycosylase: a concerted, or associative SN_2_ mechanism (Supplementary Figure S4A) and a stepwise SN_1_, or dissociative mechanism (Supplementary Figure S4B).

The DNA cleavage and glycosylase reactions by R.PabI require a large molar ratio of the enzyme to the substrate DNA. Further analysis is necessary to find out whether the enzyme turns over or not.

### Unusual features explained

The R.PabI DNA glycosylase-AP lyase pathway explains several unusual features of R.PabI in DNA cleavage. The first is that R.PabI does not require a divalent metal cation for cleavage (17). Among restriction enzymes, this feature is shared only by phospholipase D superfamily (39,40). However, this feature is characteristic of known DNA glycosylases with associated AP lyase activity (41). The second feature is difficulty in religation of the R.PabI cleavage products (Supplementary Figure S3). We previously reasoned that the 3′ end generated by R.PabI was a TA_OH_-3′ overhang (16), which appeared to be unique to the PabI family among the known restriction enzymes (2), but this needs revision based on the present findings. The 3′-blocking 4-hydroxypentenal end and the 5′-P end made by R.PabI-catalyzed β-elimination (Supplementary Figure S4C (x)) cannot be ligated by DNA ligase.

### Biological significance

Base excision and associated strand cleavage are often linked with DNA repair instead of DNA inactivation. We demonstrated that these activities can inactivate DNA. Base excision activity related to DNA inactivation has been previously observed. For example, UNGs that excise uracil from DNA (41) restrict propagation of bacteriophage carrying uracil in place of thymine in genomic DNA (42), while DNA carrying thymine (that is a methylated form of uracil) is not attacked. This process is similar to RM systems although UNG-mediated reactions involve genetic rather than epigenetic methylation. UNGs excise uracil generated by cytidine deaminase action on viral and cellular DNA (43) and generate a recombinogenic break (44), which is reminiscent of recombination stimulation by restriction endonucleases (restriction phosphodiesterases) (45).

Our finding may provide a link between DNA methylation processes in prokaryotes and eukaryotes, in a mechanistic sense and possibly in an evolutionary sense, because active demethylation in plants and animals are mediated by excision of methylated base (5-methylcytosine) or its oxidized derivative (40,41). Although little is known about the frequency with which such base excision leads to strand breakage and cell death (46,47), one such enzyme, human homolog of MutY DNA glycosylase, generates single-strand breaks and triggers cell death (48).

Our findings of a methyl-sensitive DNA glycosylase and AP lyase activities associated with a restriction-modification system expand our understanding of genetic and epigenetic processes.

## SUPPLEMENTARY DATA

Supplementary Data are available at NAR Online.

SUPPLEMENTARY DATA
