# Supplementary material for: Restriction-modification system with methyl-inhibited base excision and abasic-site cleavage activities
Source: Nucleic Acids Res. 2015 Feb 19;43(5):2841–52. doi: 10.1093/nar/gkv116 (PMC4357717; doi:10.1093/nar/gkv116)
Supplement: SUPPLEMENTARY DATA [file supp_43_5_2841__index.html]

Restriction-modification system with methyl-inhibited base excision and abasic-site cleavage activities — Restriction-modification system with methyl-inhibited base excision and abasic-site cleavage activities — SUPPLEMENTARY DATA 

# Restriction-modification system with methyl-inhibited base excision and abasic-site cleavage activities

## SUPPLEMENTARY DATA

**Files in this Data Supplement:**

- SI Table and Figure Legends
