# Supplementary material for: Restriction-modification system with methyl-inhibited base excision and abasic-site cleavage activities
Source: Nucleic Acids Res. 2015 Feb 19;43(5):2841–52. doi: 10.1093/nar/gkv116 (PMC4357717; doi:10.1093/nar/gkv116)
Supplement: SUPPLEMENTARY DATA [file supp_gkv116_nar-01554-f-2014-File007.pdf]

SI Table and Figure Legends

Table S1. *E. coli* strains and plasmids used.

Table S2. Oligonucleotides used as substrates.

Figure S1. Plasmids.

A. pBAD30_*cviQIM*. The 5'-GTAC-3' is the recognition sequence of R.PabI and M.CviQM. *cviQIM* has a ParaBAD promoter inducible with arabinose.

B. pYF46. Numbers indicate coordinates in bp.

Figure S2. Inhibition of strand cleavage and glycosylase activity of R.PabI by methylation.

A 40-mer double-stranded oligonucleotide (GTAC40 or GTAC40_full_met (Table S2), 1 pmol (100 nM)) with a ^{32}P-label at the 5' end on both strands or a 40-mer single-stranded oligonucleotide (GTAC40T, 1 pmol (100 nM)) with a ^{32}P-label at the 5' end was incubated with R.PabI (9.2 pmol (920 nM)) in sodium phosphate buffer (pH 6.5) at 37 °C or 85 °C for 3 h, treated with or without 0.1 M NaOH for 10 min at 70 °C, and separated by 10% denaturing PAGE. The supershifted bands near the top of the gel are likely DNA-R.PabI complexes (see also Fig. 5 and related text).

Figure S3. Difficulty in ligation of DNA ends generated by R.PabI.

pUC19 DNA (5.1 pmol) was cleaved with R.PabI (1.2 pmol) in 10 µl of 50 mM Tris-HCl (pH 7.5), 100 mM NaCl, 10 mM $MgCl_2$ and 1 mM DTT at 85 ^{o}C or with 10 U of R.RsaI (New England Biolabs) in 10 µl of 1x NEBuffer (10 mM Bis-tris Propane-HCl (pH 7.0), 10 mM $MgCl_2$, 1 mM DTT) at 37 ^{o}C followed by DNA purification with MagExtracter kits (TOYOBO) and ligation with Ligation High T4 DNA ligase (TaKaRa). DNA treated with the ligase was purified and cleaved again with R.RsaI. Aliquots from each step were separated by 1% agarose gel. M, DNA markers. P, product DNA.

Figure S4. Hypothetical reactions catalyzed by R.PabI.

A. DNA glycosylase: SN2 (associative) mechanism.

B. DNA glycosylase: SN1 (dissociative) mechanism.

C. AP lyase. Reduction with $NaBH_4$ is also shown. Model based on reactions of other DNA

glycosylases. Modified from (31).

Figure S5. Quantitative transformation.

Plasmid pUC19 (0.2 ng, 2 ng, 20 ng, or 200 ng) was used to transform E. coli HST08 by electroporation (Materials and Methods). Experiments were conducted in duplicate. Line, regression curve, $y = 10^{1.0308x + 4}$.

SI References

49. Yanisch-Perron, C., Vieira, J. and Messing, J. (1985) Improved M13 phage cloning vectors and host strains: nucleotide sequences of the M13mp18 and pUC19 vectors. Gene 33, 103-19.

Fig.S1

A

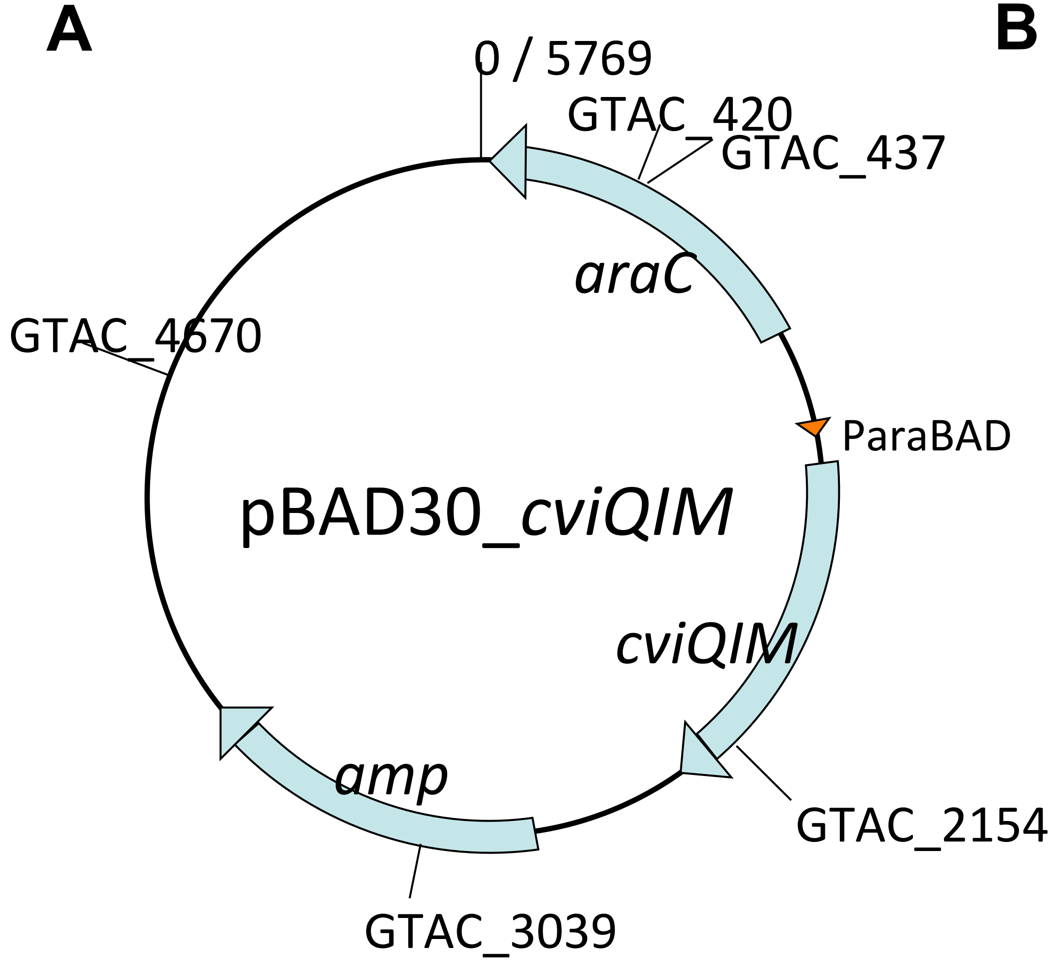
0 / 5769
GTAC_420
GTAC_437
araC
GTAC_4670
ParaBAD
pBAD30_cviQIM
cviQIM
amp
GTAC_2154
GTAC_3039

B

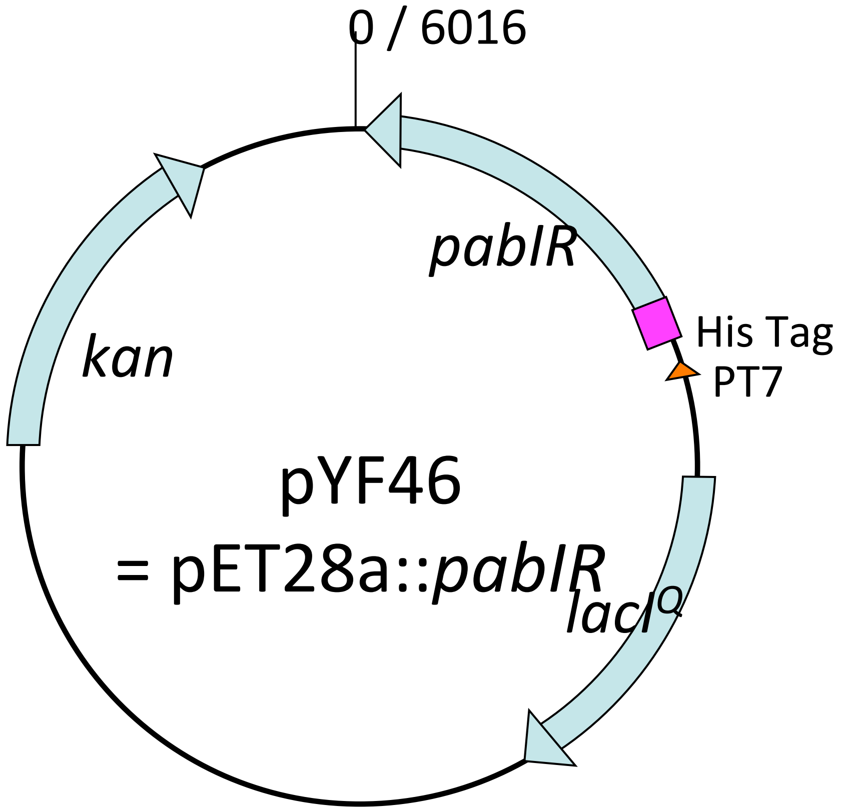
0 / 6016
pabIR
His Tag
PT7
kan
pYF46
= pET28a::pabIR
lacI^Q

Fig. S2

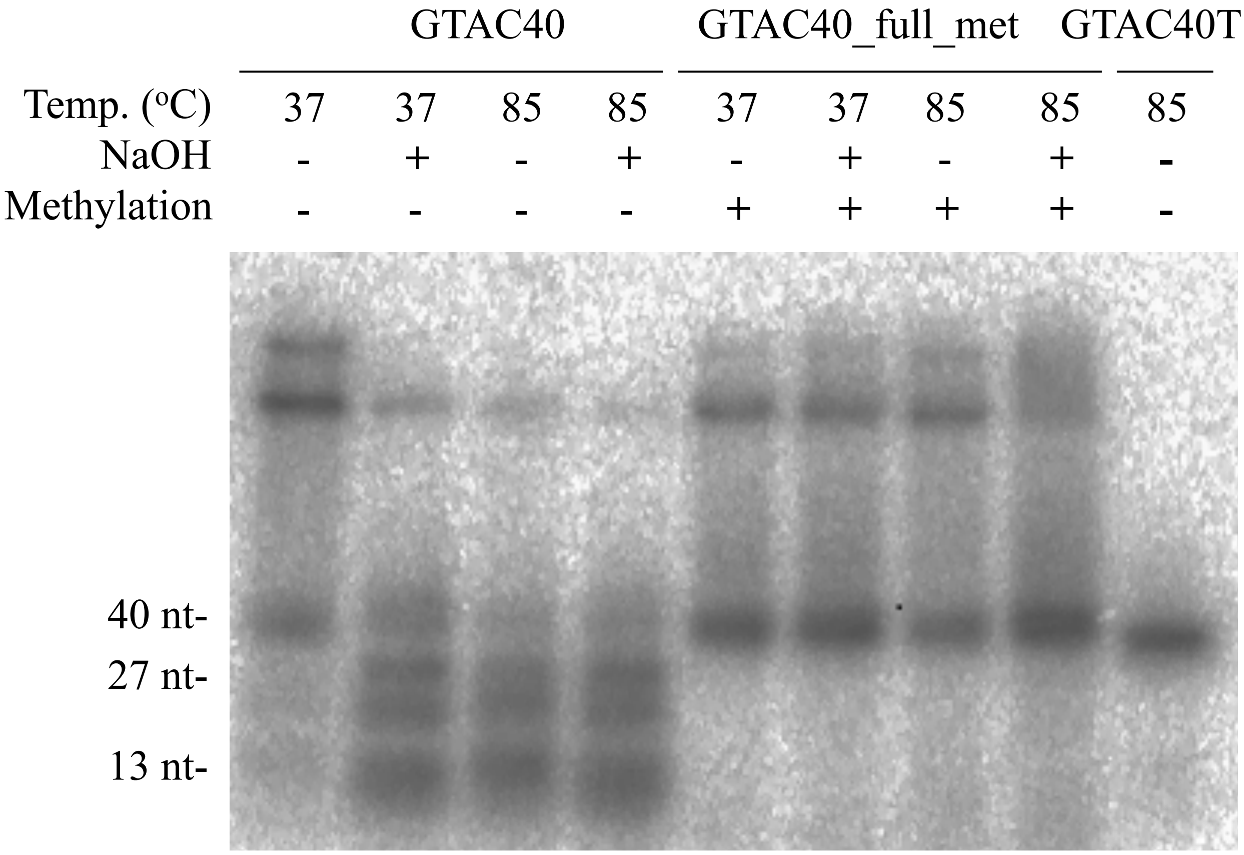
GTAC40
GTAC40_full_met
GTAC40T
Temp. (oC) 37 37 85 85 37 37 85 85 85
NaOH - + - + - + - + -
Methylation - - - - + + + + -
40 nt-
27 nt-
13 nt-

Fig. S3

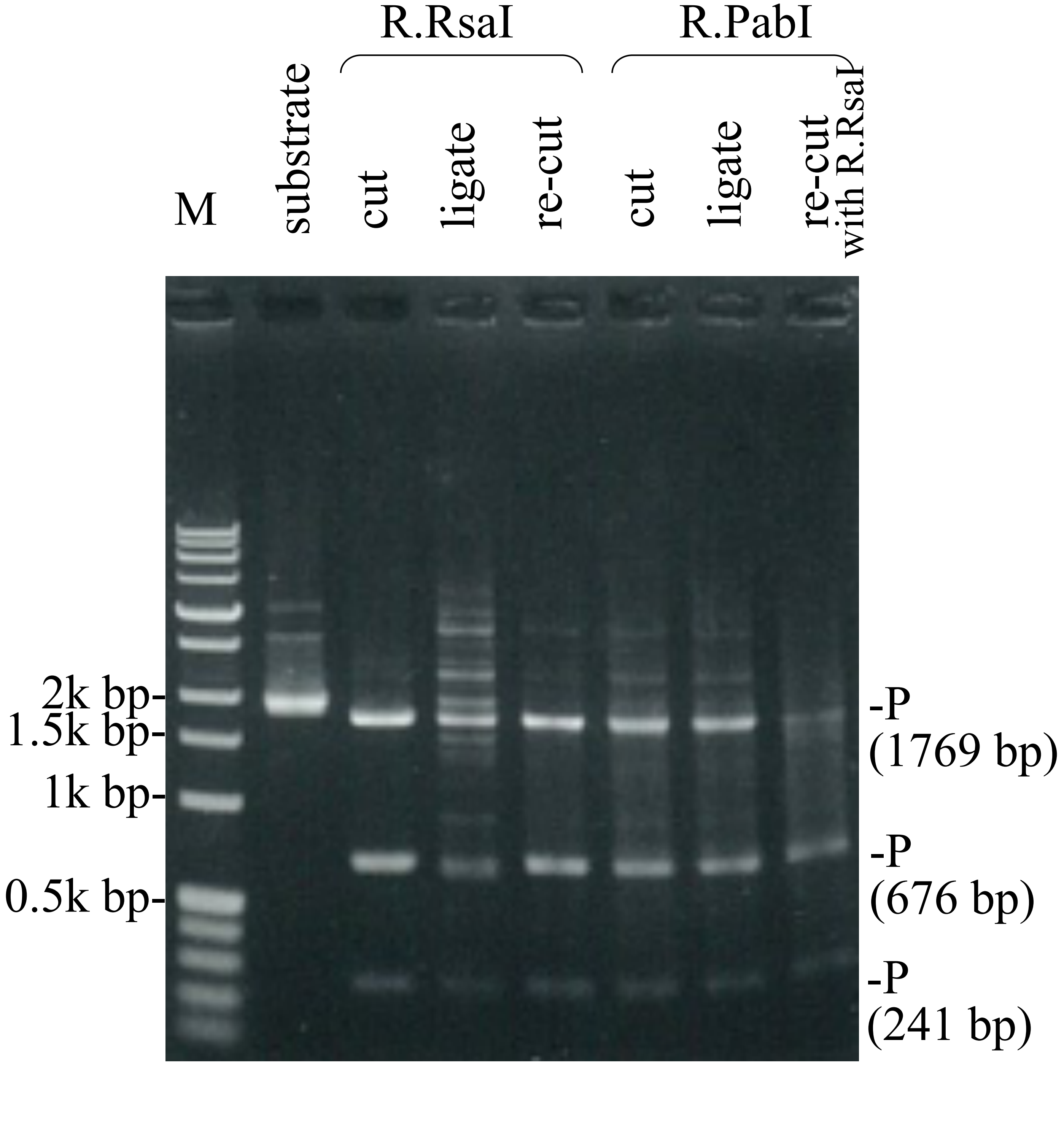
R.RsaI
R.PabI
M
substrate
cut
ligate
re-cut
cut
ligate
re-cut
with R.RsaI
2k bp-
1.5k bp-
1k bp-
0.5k bp-
-P
(1769 bp)
-P
(676 bp)
-P
(241 bp)

Fig. S4

A

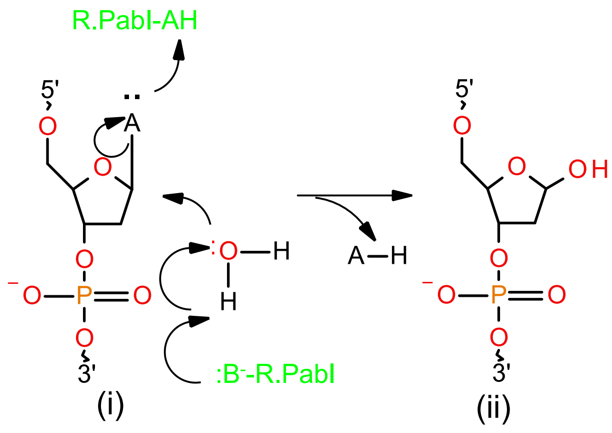

R.PabI-AH
5'
A
O
OH
O—H
H
A—H
:B⁻-R.PabI
3'
(i)
(ii)

B

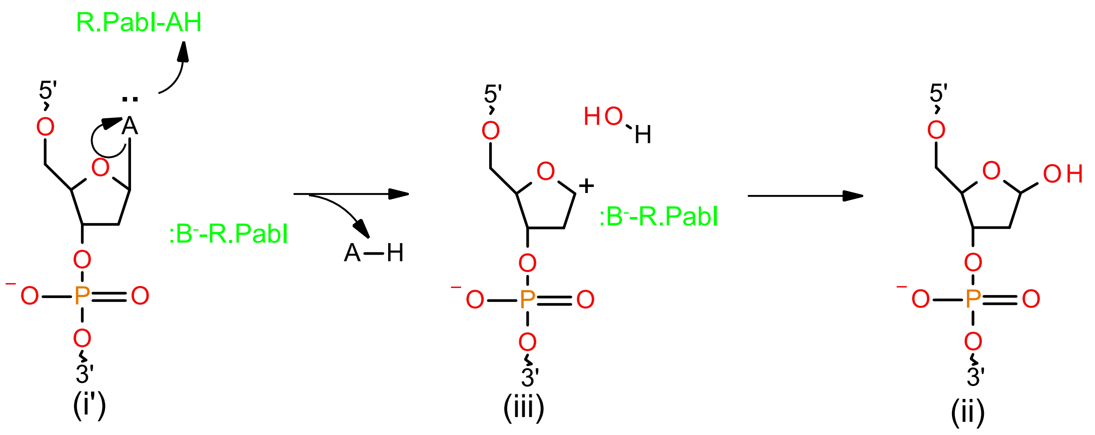

R.PabI-AH
5'
A
:B⁻-R.PabI
A—H
HO H
OH
3'
(i')
(iii)
(ii)

C

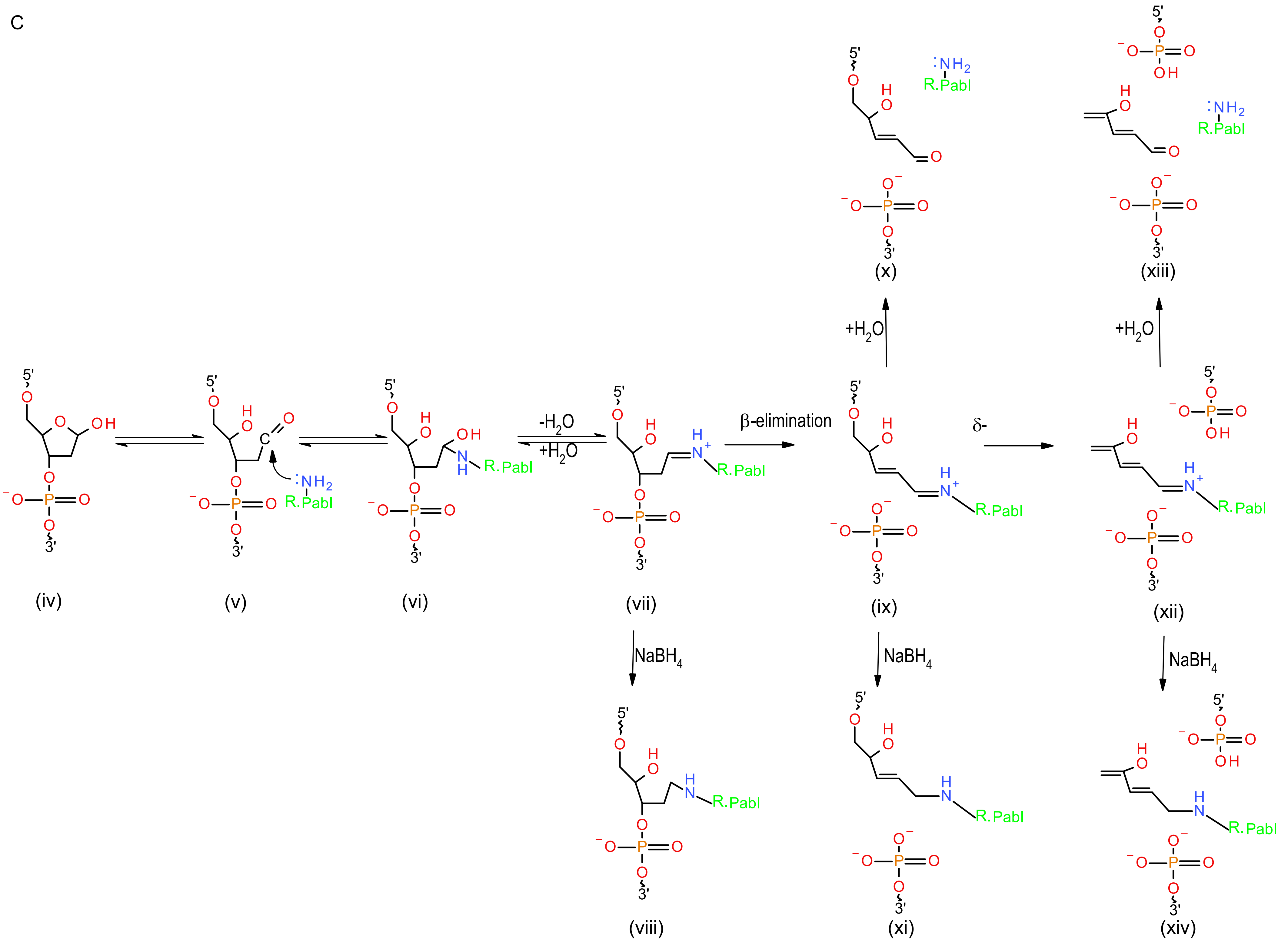

:NH2
R.PabI
-H2O
+H2O
β-elimination
δ-
NaBH4
5'
3'
(iv)
(v)
(vi)
(vii)
(viii)
(ix)
(x)
(xi)
(xii)
(xiii)
(xiv)

Fig.S5

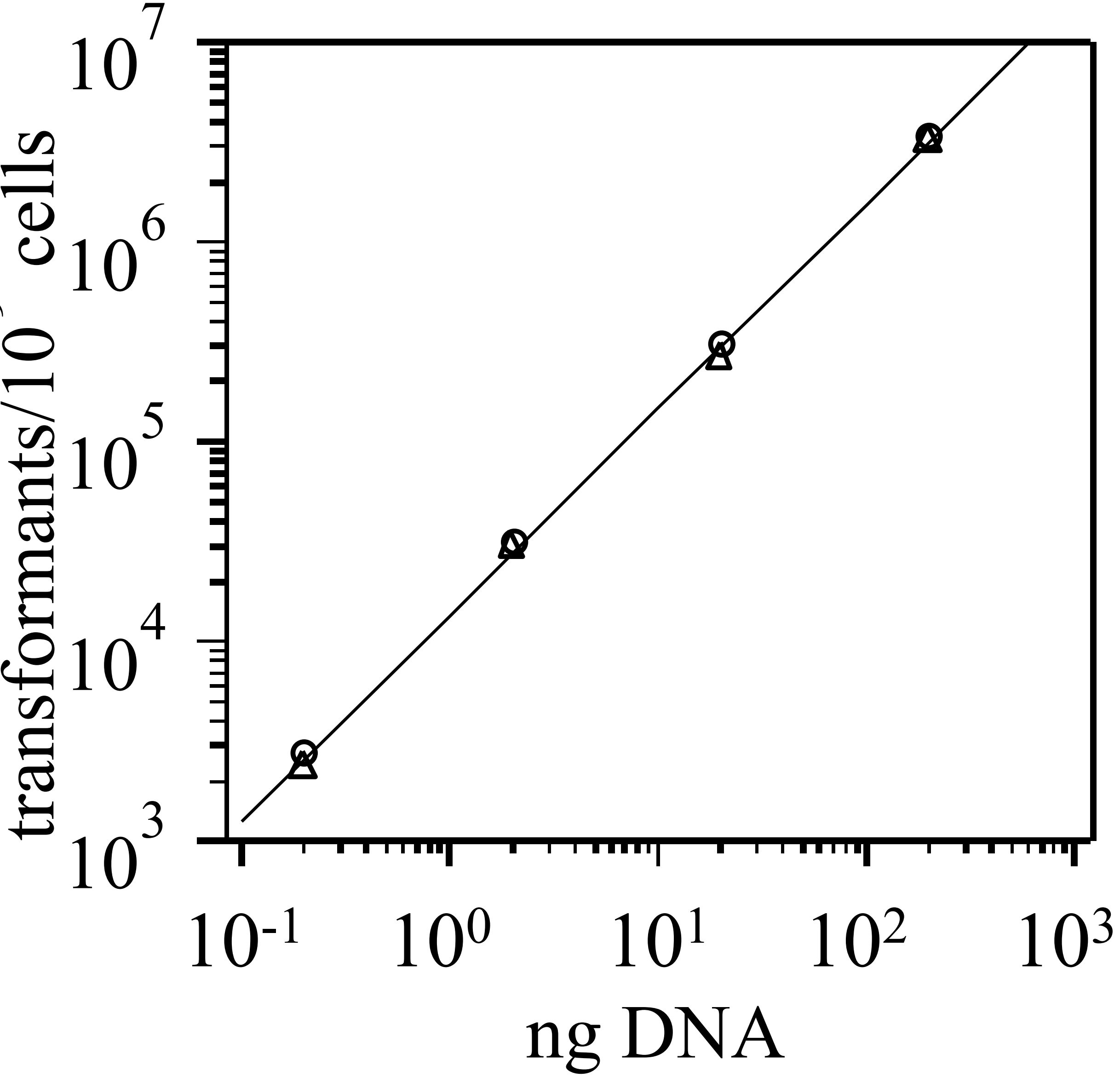

10^7
10^6
10^5
10^4
10^3
transformants/10^9 cells
10^{-1}
10^0
10^1
10^2
10^3
ng DNA

Table S1. *E. coli* strains and plasmids used

	Name	Relevant properties	Comments	Source /reference
E. coli strains	HST08	F-, *endA1, supE44, thi-1, recA1, relA1, gyrA96, phoA*, Φ80d*lacZΔM15*, Δ(*lacZYA-argF*)U169, Δ(*mrr-hsdRMS-mcrBC*), Δ*mcrA*, λ-		TaKaRa
	BMF235	HST08 (pBAD30_*cviQIM*)		This work
	BL21 (DE3)	F^{-}, *dcm, ompT, hsdS*$_B$(r_B^{-} m_B^{-}), *gal* (λDE3 = *λcI857, ind1, Sam7, nin5, lacUV5-T7gene1*)		(26)
	BYF25	BL21 (DE3) (pBAD30_*cviQIM*)		This work
	BYF72	BL21 (DE3) (pBAD30_*cviQIM*) (pET28a::*pabIR*)		This work
Plasmids	pBAD30	P_{BAD}, Cml^R		(24)
	pBAD30_*cviQIM*	pBAD30 *cviQIM*+	Fig. S1	This work
	pUC19	Amp^R		(47)
	pMW40	*pabIR*		(17)
	pET28a	P_{T7}, *lacI*^{Q}, Kan^R		Novagen, (26)
	pYF46	pET28a::*pabIR*		This work

Table S2. Oligonucleotides used as substrates.

Oligonucleotide name	Length (nt or bp)	Sequence	Source
GTAC40T	40	5′- GGGGAGGCGCCGGCAGTGCGTCAG**GTAC**TCCGCCACGTCC - 3′	Hokkaido System Science
GTAC40B	40	5′- GGACGTGGCGGA**GTAC**CTGACGCACTGCCGGCGCCTCCCC - 3′	Hokkaido System Science
GTAC40Tme	40	5′- GGGGAGGCGCCGGCAGTGCGTCAG**GTmAC**TCCGCCACGTCC - 3′	Hokkaido System Science
GTAC40Bme	40	5′- GGACGTGGCGGA**GTmAC**CTGACGCACTGCCGGCGCCTCCCC - 3′	Hokkaido System Science
GTUC40T	40	5′- GGGGAGGCGCCGGCAGTGCGTCAG**GTUC**TCCGCCACGTCC - 3′	Tsukuba Oligo Service
GTUC40B	40	5′- GGACGTGGCGGA**GTUC**CTGACGCACTGCCGGCGCCTCCCC - 3′	Tsukuba Oligo Service
GCUC40T	40	5′- GGGGAGGCGCCGGCAGTGCGTCAG**GCUC**TCCGCCACGTCC - 3′	Tsukuba Oligo Service
GCUC40B	40	5′- GGACGTGGCGGA**GCUC**CTGACGCACTGCCGGCGCCTCCCC - 3′	Tsukuba Oligo Service
ATUC40T	40	5′- GGGGAGGCGCCGGCAGTGCGTCAG**ATUC**TCCGCCACGTCC - 3′	Tsukuba Oligo Service
GTUT40B	40	5′- GGACGTGGCGGA**GTUT**CTGACGCACTGCCGGCGCCTCCCC - 3′	Tsukuba Oligo Service
GTAC40	40	5′- GGGGAGGCGCCGGCAGTGCGTCAG**GTAC**TCCGCCACGTCC - 3′<br>3′- CCCCTCCGCGGCCGTCACGCAGTC**CATG**AGGCGGTGCAGG - 5′	GTAC40T + GTAC40B
GTAC40_hemi_met	40	5′- GGGGAGGCGCCGGCAGTGCGTCAG**GTmAC**TCCGCCACGTCC - 3′<br>3′- CCCCTCCGCGGCCGTCACGCAGTC**CAT-G**AGGCGGTGCAGG - 5′	GTAC40Tme + GTAC40B
GTAC40_full_met	40	5′- GGGGAGGCGCCGGCAGTGCGTCAG**GT-mAC**TCCGCCACGTCC - 3′<br>3′- CCCCTCCGCGGCCGTCACGCAGTC**CmAT-G**AGGCGGTGCAGG - 5′	GTAC40Tme + GTAC40Bme
GT#C40	40	5′- GGGGAGGCGCCGGCAGTGCGTCAG**GT#C**TCCGCCACGTCC - 3′<br>3′- CCCCTCCGCGGCCGTCACGCAGTC**C#TG**AGGCGGTGCAGG - 5′	Treat GTUC40T/GTUC40B with UNG, # = AP site

GC#C40	40	5′- GGGGAGGCGCCGGCAGTGCGTCAG**GC#C**TCCGCCACGTCC - 3′<br>3′- CCCCTCCGCGGCCGTCACGCAGTC**C#CG**AGGCGGTGCAGG - 5′	Treat GCUC40T/GCUC40B with UNG, # = AP site
AT#C40	40	5′- GGGGAGGCGCCGGCAGTGCGTCAG**AT#C**TCCGCCACGTCC - 3′<br>3′- CCCCTCCGCGGCCGTCACGCAGTC**T#TG**AGGCGGTGCAGG - 5′	Treat ATUC40T/GTUT40B with UNG, # = AP site
GT#C40EIII	40	5′- GGGGAGGCGCCGGCAGTGCGTCAG**GT#C**TCCGCCACGTCC - 3′<br>3′- CCCCTCCGCGGCCGTCACGCAGTC**CATG**AGGCGGTGCAGG - 5′	Treat GTUC40T/GTAC40B with UNG, # = AP site
